# Diversity of volatile phenols and their newly identified precursors during different grape species development

**DOI:** 10.3389/fpls.2026.1746115

**Published:** 2026-03-11

**Authors:** Ziang Zheng, Weixi Yang, Mengyao Qi, Yanfang Bai, Qingsen Kong, Dongqing Ye, Guo Cheng, Keji Yu, Changqing Duan, Yibin Lan

**Affiliations:** 1 Key Laboratory of Viticulture and Enology, Ministry of Agriculture and Rural Affairs, College of Food Science & Nutritional Engineering, China Agricultural University, Beijing, China; 2 Ji’an Yajianggu Winery Co., Ltd., Tonghua, China; 3 Guangxi Key Laboratory of Fruits and Vegetables Storage-Processing Technology, Guangxi Academy of Agricultural Sciences, Nanning, Guangxi, China; 4 Grape and Wine Research Institute, Guangxi Academy of Agricultural Sciences, Nanning, China

**Keywords:** East Asian species, glycosidic precursors, grape development, pseudo-targeted metabolomics, volatile phenols

## Abstract

Grape-derived volatile phenols significantly contribute to wine aroma, yet their developmental dynamics and species-specific regulation in grapes remain poorly understood. This study monitored 18 volatile phenols and identified 20 glycosidic precursors during berry development in three East Asian grape species (*Vitis davidii*, *V. quinquangularis*, *V. amurensis*) and *V. vinifera* cv. Cabernet Sauvignon from two regions (Ningxia and Changli). Using a pseudo-targeted metabolomic approach coupled with UHPLC-qTOF-MS/MS, we profiled precursors of *m/p*-cresol, guaiacol, 4-vinyl-phenol, 4-vinyl-guaiacol, isoeugenol, and coniferaldehyde. Time-series analysis revealed markedly distinct accumulation patterns: East Asian species showed progressive accumulation of volatile phenols and higher precursor concentrations than *V. vinifera*, with *V. davidii* exhibiting the highest levels. Cabernet Sauvignon displayed contrasting regional patterns—gradual accumulation in Changli versus an early peak followed by decline in Ningxia. Six precursors were exclusive to East Asian species, indicating species-specific glycosyltransferase activities and serving as potential markers for predicting varietal identity. Widespread precursors (e.g., glycosidic *m/p*-cresol, isoeugenol, and coniferaldehyde) showed strong positive correlations with their volatile phenols, demonstrating predictive potential. Collectively, these results provide a systematic analysis of the expression of volatile phenols and their precursors and reveal pronounced metabolic differences between East Asian and *V. vinifera* grapes, as well as between growing regions. This knowledge provides actionable guidance for vineyard management, varietal selection, and wine style development.

## 1 Introduction

Volatile phenols are an important class of aroma compounds commonly found in wine, including phenol, guaiacol, syringol, and their derivatives formed by the substitution of hydrogen atoms on the benzene ring by alkanes (Yang, 2024). Certain volatile phenols, such as cresol, guaiacol, eugenol, isoeugenol, and syringol, possess low sensory thresholds in wine (Boidron et al., 1988; Waterhouse et al., 2016), and previous studies have reported their concentrations in some wines far exceeding their thresholds (Kennison et al., 2007), directly contributing to the aroma of wine (Yang, 2024). Generally, volatile phenols that naturally occur in grape berries or are extracted through well-managed processes such as oak aging do not impair the aromatic quality of wine. Among these volatile phenols, compounds such as vanillin, eugenol, and guaiacol contribute to vanilla, woody, and smoky aromas, enhancing the overall complexity of the wine’s flavor profile (Cameleyre et al., 2020). Meanwhile, volatile phenols interact with other aroma compounds and can influence aroma perception even when their concentrations are below sensory thresholds (Cameleyre et al., 2020). Low concentrations of vanillin can significantly enhance fruity aromas in model wines, whereas compounds such as guaiacol, 4-ethylphenol, and *o*-cresol can diminish the fruitiness in wine (Cameleyre et al., 2020; McKay et al., 2021). The masking effect of these volatile phenols on fruitiness can, in some cases, compromise the overall aroma of wine. Some studies have reported high levels of volatile phenols, mainly in contaminated wines or in specific grape varieties such as East Asian species (Favell et al., 2022; Yang et al., 2023). For example, excessive levels of cresols and guaiacol may lead to undesirable aromas, including ash, burnt, or medicinal notes (Parker et al., 2012), which can significantly reduce consumer appreciation of the wine. Study has also found that interactions between volatile phenols and 2−iso−butyl−3−methoxypyrazine (IBMP) can generate undesirable burnt aromas (McKay et al., 2021). Due to their dual sensory properties, volatile phenols serve as critical markers for grape and wine quality assessment. In addition to their role in flavor formation, certain volatile phenols, such as methyl eugenol, serve critical functions in plant defense mechanisms against herbivory and insect damage, while also mediating insect-guided pollination (Tan and Nishida, 2012). Nevertheless, due to their phytotoxicity, their accumulation triggers a glycosylation response, mitigating potential harm to plant tissues (Song et al., 2018).

Volatile phenols in wine originate from four primary sources: wildfires smoke, *Dekkera/Brettanomyces* spoilage, oak aging, and endogenous grape metabolism (Summerson et al., 2021; Milheiro et al., 2019; Waterhouse et al., 2016; Yang et al., 2025). A recent study reported that East Asian wines exhibited high concentrations of volatile phenols, which are derived from the fruit’s endogenous production (Yang et al., 2023), and nine glycosides of eugenol and syringol were identified as key fruit-derived precursors which were abundant in East Asian species (Yang et al., 2025). These aroma compounds and their precursors in the fruit are also influenced by the grape variety and the growing region. Eugenol, syringol, 4−ethylphenol, and 4−ethylguaiacol and their precursors were more abundant in wines produced from East Asian grape species (Yang, 2024).

Beyond East Asian species, volatile phenol profiles show strong varietal dependence. Red grapes generally accumulate higher concentrations than white varieties, with Syrah exhibiting particularly elevated guaiacol (Coulter et al., 2022). Syringol gentiobioside follows a cultivar-specific pattern, being highest in Shiraz, intermediate in Grenache, Mataro and Merlot, and the lowest in Pinot Noir and Sangiovese (Coulter et al., 2022). The vineyard environment has a significant impact on grape aroma (Fischer et al., 1999); however, studies specifically addressing volatile phenols are relatively limited. Most studies have examined wildfire smoke impacts across regions (Mirabelli-Montan et al., 2021; Tan et al., 2024). Water availability represents another key influence, as low water uptake has been associated with increased concentrations of bound volatile phenols in grapes (Koundouras et al., 2006).

In plants, the phenylpropanoid pathway, which originates from phenylalanine, biosynthesizes volatile phenols including eugenol, isoeugenol, chavicol, isochavicol, methyleugenol, methylisoeugenol, estragole, and anethole (Yang, 2024). Eugenol and isoeugenol biosynthesis share initial steps with lignin biosynthesis. After the formation of *p*-coumaryl alcohol and coniferyl alcohol, coniferyl alcohol acyltransferase (CFAT) converts them to their acetate esters, which are subsequently reduced by phenylpropene reductase (PhR) to yield eugenol and isoeugenol (Atkinson, 2018; Koeduka et al., 2006). In addition, volatile phenols such as guaiacol, methylguaiacol, cresol, vinylguaiacol, and methylsyringol have also been identified in grape berries (Coulter et al., 2022; Meng et al., 2013; Noguerol-Pato et al., 2012). These compounds predominantly occur in grapes as glycosidically bound forms, including monoglucosides, gentiobiosides, pentosylglucosides, rutinosides, and trisaccharides, with disaccharide glycosides being the predominant type (Hayasaka et al., 2013; Caffrey et al., 2019). These glycosidically-bound volatile phenols gradually accumulate during development or undergo dynamic changes (Noestheden et al., 2018). Szeto et al. (2020) reported a general increase in glycosylated guaiacol, 4-methylguaiacol, phenol, cresol, syringol, and 4-methylsyringol after véraison, while glycosylated guaiacol specifically has been shown to exhibit a rise-and-fall pattern in some cultivars (Dungey et al., 2011). These glycosidic precursors serve as a key reservoir of aroma-active compounds that can be hydrolyzed during winemaking (Caffrey et al., 2021). Although they make no direct sensory contribution, their hydrolysis, via enzymatic or acid-catalyzed pathways, exerts a significant influence on the development of wine flavor (Kennison et al., 2008). Despite their importance, the developmental dynamics of volatile phenol precursors in grapes remain poorly understood, limiting our ability to predict or regulate their final concentrations in wines.

This study was designed to systematically elucidate the developmental dynamics of volatile phenols and their glycosidic precursors in grape berries. Our specific objectives were threefold: (1) to quantify volatile phenols throughout berry development and identify compounds with potential contributions to aroma, including *m/p*-cresol, guaiacol, 4-vinylphenol, 4-vinylguaiacol, isoeugenol, and coniferaldehyde; (2) to identify their corresponding glycosidic precursors using an integrated correlation analysis and pseudo-targeted metabolomic approach; and (3) to characterize the temporal accumulation patterns of these precursors across different grape species and growing regions.

## 2 Materials and methods

### 2.1 Grapes

Grape berries from four cultivars were collected at different developmental stages from various regions in China. Among them, three cultivars belong to East Asian species, including Tian grape (*V. davidii*) from Hunan Province, Yeniang No. 2 (*V. quinquangularis*) from Guangxi Province, and Shuanghong (*V. amurensis*) from Jilin Province. Additionally, two Cabernet Sauvignon (*V. vinifera*) grape samples were sampled from Ningxia and Changli region, respectively. Grape harvest dates, cultivation practices, and planted years are listed in **Supplementary Table 1**. Temperature and precipitation data were obtained from the China Meteorological Data Center (https://data.cma.cn; **Supplementary Table 2**). At harvest, ten clusters without physical damage were randomly collected from both sides of the canopy of ten different vines, and 300 berries were sampled from the top, bottom, and middle of each cluster. All grape samples were immediately frozen at -40°C after sampling.

### 2.2 Chemicals

Sodium chloride, sodium hydroxide, glucose, anhydrous copper sulfate, citric acid, disodium hydrogen phosphate, and potassium hydrogen phthalate (analytical grade) were purchased from Beijing Chemical Works (Beijing, China). HPLC-grade formic acid was purchased from ROE Scientific (Newark, NJ, USA). Chromatographic grade solvents, including acetonitrile, methanol, ethanol, and dichloromethane, were purchased from Honeywell (Morris Township, NJ, USA). Reference standards of aroma compounds and C_6_-C_24_*n*-alkanes were purchased from Sigma-Aldrich (St. Louis, MO, USA). Rapidase AR2000 was purchased from DSM Food Specialties (Delft, Netherlands).

### 2.3 Determination of free and bound volatile phenols by SPE-GC-QqQ-MS/MS

Grape juice extraction was performed based on a previously reported method with slight modifications (Lan et al., 2019). Weigh out 50 g of grape berries stored at -80°C, remove the stems and seeds under liquid nitrogen protection. Then, 0.5g of D-glucono-δ-lactone and 1g of PVPP were added. The berries were ground into a fine powder using a fruit grinder. The resulting powder was soaked at 4°C in a sealed container for 4hours, and then centrifuged at 8000rpm for 10minutes at 4°C. The clarified supernatant was collected as grape juice.

The analysis of free volatile phenols was carried out following a previously reported method (Yang et al., 2025). Volatile phenols were extracted using solid-phase extraction (SPE) with a Cleanert PEP-SPE cartridge (500 mg/6 mL, Bonna-Agela Technologies, China). Before extraction, the cartridge was conditioned sequentially with 10 mL dichloromethane, 10 mL methanol, and 10 mL Milli-Q water. A 10 mL sample was spiked with 10 μL of internal standard (3,4-dimethylphenol, 0.2 g/L in ethanol) and diluted with 10 mL of deionized water, then loaded at a flow rate of 1 mL/min. To remove sugars and polar components, the column was rinsed with 5 mL Milli-Q water. Volatile compounds were eluted using 10 mL of dichloromethane. The volatile phenol precursors were subsequently eluted using 10 mL of methanol. The dichloromethane fraction was concentrated to 1 mL under a gentle stream of nitrogen for the determination of free volatile phenols by GC-QqQ-MS/MS.

Hydrolysis assays were performed to analyze bound volatile phenols. The methanol extract of volatile phenol precursors was evaporated to dryness under vacuum at 30°C using a rotary evaporator. The residue was then reconstituted in 10 mL of citrate-phosphate buffer (pH 3.0), followed by the addition of 200 μL of AR2000 enzyme solution (100 g/L). The enzymatic hydrolysis was carried out at 40°C for 16 hours. Subsequently, volatile phenols were re-extracted from the hydrolyzed samples using the same solid-phase extraction method described earlier. Bound volatile compounds were eluted using 10 mL of dichloromethane. The dichloromethane fraction was concentrated to 1 mL under a gentle stream of nitrogen for the determination.

Quantification of volatile phenols was conducted using an Agilent 7890B gas chromatograph coupled with a 7000D triple quadrupole mass spectrometer (GC-TQ, Agilent Technologies, Palo Alto, CA, USA). Separation was achieved on an HP-5MS UI capillary column (30m × 250μm × 0.25μm, Agilent Ultra Inert). A 1 μL aliquot of the concentrated dichloromethane extract was introduced in split mode (4:1). Helium served as the carrier gas at 1.0mL/min, with an additional 2.25 mL/min as quenching gas, while nitrogen was used as the collision gas at a rate of 1.5 mL/min. Instrument temperatures were set as follows: injector at 250°C, ion source at 230°C, transfer line at 300°C, and both quadrupoles at 150°C. The oven program initiated at 40 °C, ramping to 80°C at 5 °C/min, then to 120°C at 2 °C/min, further to 160°C at 5 °C/min, and finally to 220°C at 30 °C/min. A post-run at 280°C for 1minute was applied after each analysis. The mass spectrometer operated under electron ionization (EI) at 70 eV, and quantification was carried out in multi-reaction monitoring (MRM) mode (details in **Supplementary Table 3**).

### 2.4 Identification and determination of the precursors of volatile phenols by UHPLC-qTOF-MS/MS

Identification of the precursors was achieved by UHPLC-qTOF-MS/MS using targeted MS/MS mode. The peak areas of the predicted precursors were obtained from the full-scan data, from which the reduction in quantity after hydrolysis was calculated. Correlation analysis of bound volatile phenols and the reduction of potential precursors were performed using Pearson’s method (P < 0.05 and correlation coefficient ≥ 0.5). The predicted precursors with high correlation were qualitatively analyzed using targeted MS/MS mode. The predicted volatile phenol precursors were determined in the samples both prior to and following enzymatic hydrolysis. To this end, two sets of samples were prepared: one was enzymatically hydrolyzed using the method described above, while the other remained non-hydrolyzed. All of samples were then extracted using solid-phase extraction (SPE) following the procedure outlined in section 2.3 for subsequent analysis. The methanol extract was evaporated to dryness under vacuum at 30°C using a rotary evaporator. Afterward, the dried methanol extract was redissolved in 2 mL of a reconstitution solution (5% acetonitrile aqueous solution) for the direct analysis of the precursors of volatile phenol by UHPLC-qTOF-MS/MS.

A further analysis was performed to determine the precursors of volatile phenols. This included both the precursors of 4-ethylphenol, 4-ethylguaiacol, eugenol, and syringol reported previously (Yang, 2024) and those identified in the current study (**Supplementary Table 4**). UHPLC-qTOF analysis was performed on an Agilent 1290II series ultra-high performance liquid chromatography (UHPLC) coupled to a 6454 quadrupole time of flight mass spectrometer (qTOF). Separation was performed on an InfinityLab Poroshell 120SB-C18 column (2.1 × 150 mm, 2.7 μm) maintained at 50°C. The mobile phases consisted of 0.1% formic acid in water (phase A) and 0.1% formic acid in acetonitrile (phase B), with a flow rate of 0.4 mL/min. A 5 μL sample was injected for analysis. The elution profile was as follows: 5-10% B from 0 to 12 min, 10-40% B from 12 to 14 min, and 40-100% B from 14 to 16 min.

Full-scan mass spectrometry was conducted using a Dual AJS ESI ion source operated in negative ion mode. The scan range was set from m/z 70 to 1700, with a data acquisition rate of 2 spectra per second. Nitrogen served as both the sheath and drying gases, delivered at 10 L/min and 8 L/min respectively, both maintained at 350°C. The atomizing gas pressure was set at 60 psig. The nozzle and capillary voltages were adjusted to 1 kV and 3.5 kV, respectively. Mass calibration in negative ion mode employed a standard tuning solution (G1969-85000, Supelco Inc.), using reference ions TFANH_4_ (112.9856 m/z) and HP 0921 (1033.9881 m/z), with an allowable mass error within ±0.2 ppm.

Target MS/MS analysis was performed using a Dual AJS ESI ion source operated in negative ion mode. The mass spectrometry parameters were identical to those described above. The method relied on targeted acquisition, using the exact mass of precursor ions, retention times, and optimized fragmentation voltages as key parameters. Qualitative identification of compounds was based on comparison of retention times and mass spectral data. Identification was further confirmed by UHPLC-qTOF analysis through accurate mass detection and interpretation of corresponding MS/MS fragmentation patterns.

### 2.5 Statistical analysis

Pearson’s method was performed by Graphpad Prism 9.5.1 to identify potential precursors with high correlation. Analysis of variance (ANOVA) was performed using Tukey’s test at a significance level of p < 0.05 (SPSS 26). Time series analysis was performed by using ‘C-means analysis’ in the ‘mfuzz’ package in R statistical environment (4.4.1) to explore the developmental trends of volatile phenols and their precursors.

## 3 Result and discussion

### 3.1 Concentration and developmental patterns of volatile phenols in grapes

#### 3.1.1 The volatile phenols in ripen grapes

**Figure 1:**
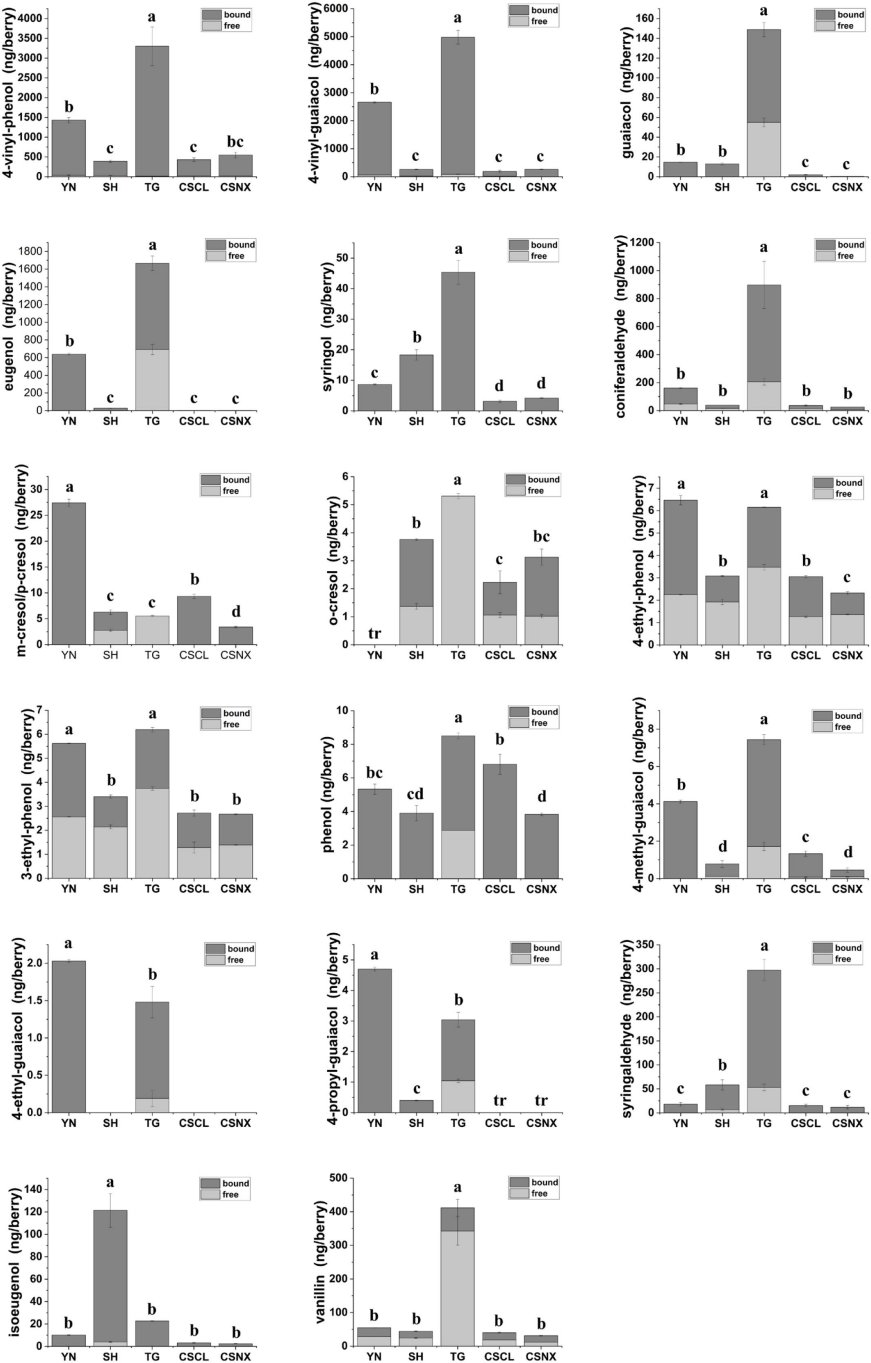
Concentration of volatile phenols in mature grapes; Different lower-case letters indicated that there were significant differences between different treatment groups at the mature grape (Turkey, p<0.05); CSNX, Cabernet Sauvignon from Ningxia; CSCL, Cabernet Sauvignon from Changli; SH, Shuanghong; TG, Tian grape; YN, Yeniang No. 2.

Eighteen volatile phenols in grapes were quantitatively analyzed, including both free and bound forms detected by GC-QqQ-MS/MS. To investigate their variation patterns during berry development, juice yield and berry weight were measured (**Supplementary Table 5**), and the concentrations of volatile phenols were converted to content per berry (**Supplementary Table 6**). The volatile phenol concentrations in mature grapes are presented in **Figure 1**.

The highest levels of 4-vinyl-phenol and 4-vinyl-guaiacol were observed in mature grapes (**Figure 1**), with both compounds predominantly present in bound forms. Bound 4-vinyl-phenol accounted for 91.97 - 99.59% of the total concentration, while bound 4-vinyl-guaiacol accounted for 91.73 - 98.45%. The highest concentrations of both vinyl-phenols were found in Tian grapes, followed by Yeniang No. 2. The lowest levels were found in Shuanghong and Cabernet Sauvignon grapes from both regions. While guaiacol, eugenol, syringol and coniferaldehyde were most abundant in Tian grapes, Yeniang No.2 was characterized by higher concentrations of *m*/*p*-cresol, key odorants associated with smoky, ashy, and medicinal notes (Huo et al., 2024). Guaiacol is an endogenous constituent of grapes (Yang et al., 2023), though its biosynthetic origin remains uncertain. Recent studies propose two potential mechanisms: lignin degradation during berry development (Wang et al., 2025) and enzymatic biosynthesis via catechol−*O*−methyltransferase (Mageroy et al., 2012).

It was found that isoeugenol is the predominant volatile phenol in Shuanghong, distinguishing it from the others. Both eugenol and isoeugenol are biosynthesized via the phenylpropanoid pathway (Koeduka et al., 2006). The elevated volatile phenol levels across East Asian species thus reflect more active secondary metabolism in their berries, forming the biochemical basis for their distinctive varietal aromas.

The concentrations of phenol, *o*-cresol, 4-ethyl-phenol, 3-ethyl-phenol, 4-methyl-guaiacol, 4-ethyl-guaiacol, and 4-propyl-guaiacol in grapes were all well below sensory threshold in wine. This observation is consistent with previous findings that phenol, cresols, and 4-methylguaiacol are mainly associated with smoke-tainted wines (Szeto et al., 2024), while 4-ethylphenol and 4-ethylguaiacol are generally produced through microbial metabolism (Chatonnet et al., 1997). In ripen grapes, other volatile phenols were also found to be primarily in bound forms. The proportion of bound forms for compounds such as guaiacol, eugenol, isoeugenol, syringol, and coniferaldehyde ranged from 58.51% to 100%. These results highlight those bound forms of aroma compounds in grapes play a fundamental role in shaping the final aroma profile of wine (He et al., 2023). Nevertheless, the specific structures of these bound volatile phenols still require further identification.

#### 3.1.2 The evolution of volatile phenols in grape development

**Figure 2:**
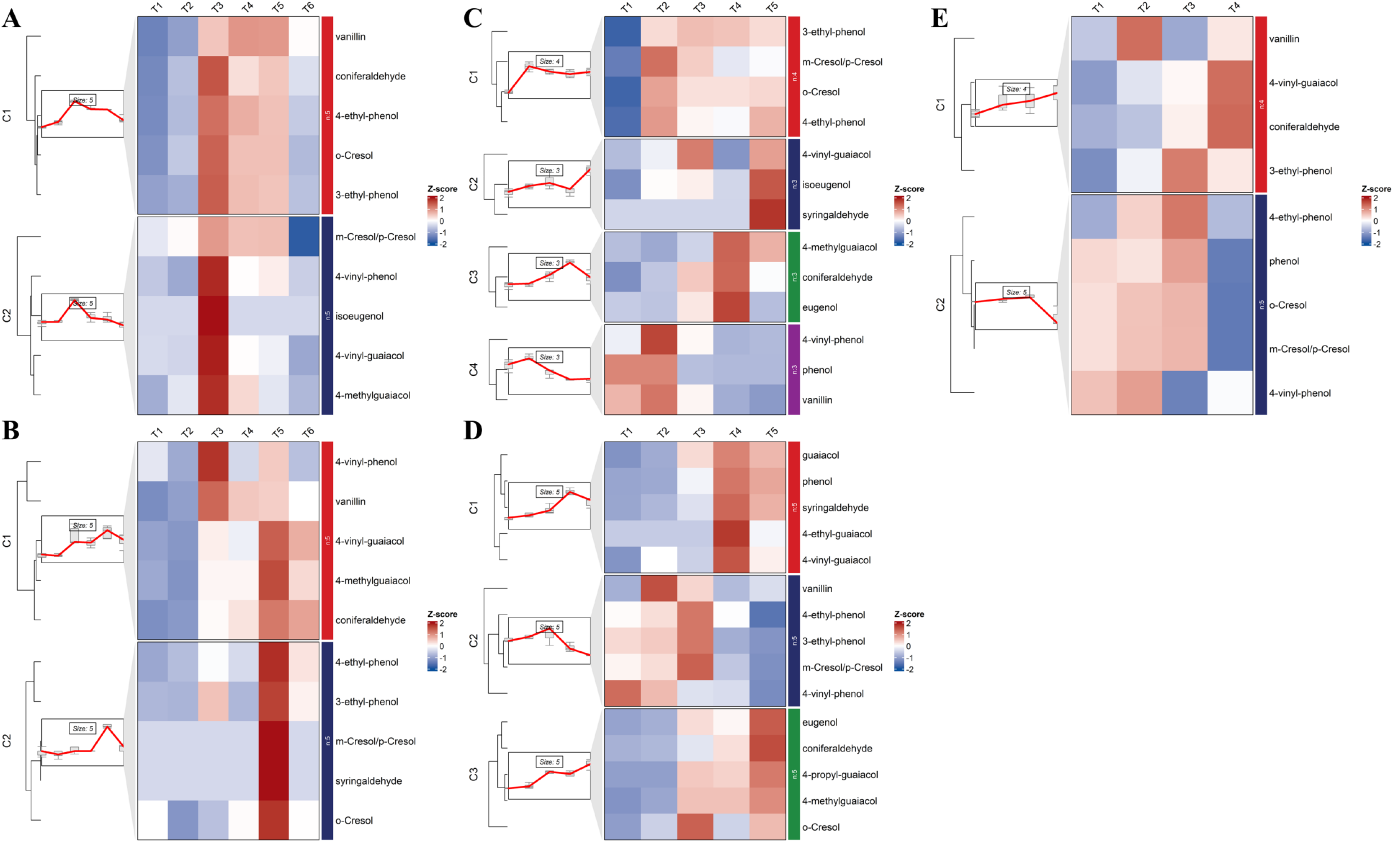
Time series analysis of free volatile phenols during the development of Cabernet Sauvignon from Ningxia **(A)**, Cabernet Sauvignon from Changli **(B)**, Shuanghong **(C)**, Tian grape **(D)**, and Yeniang No.2 **(E)**.

**Figure 3:**
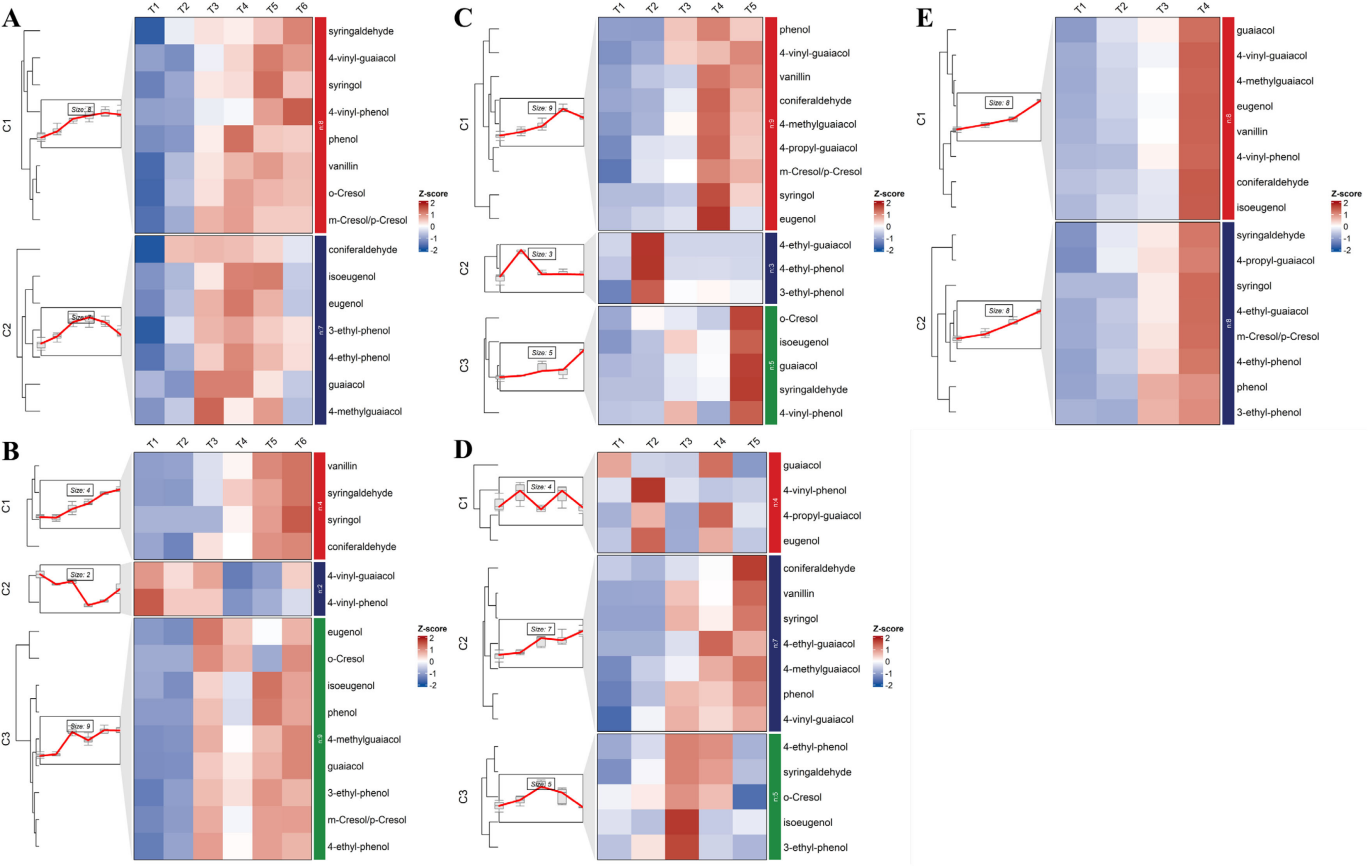
Time series analysis of bound volatile phenols during the development of Cabernet Sauvignon from Ningxia **(A)**, Cabernet Sauvignon from Changli **(B)**, Shuanghong **(C)**, Tian grape **(D)**, and Yeniang No.2 **(E)**.

**Figure 4:**
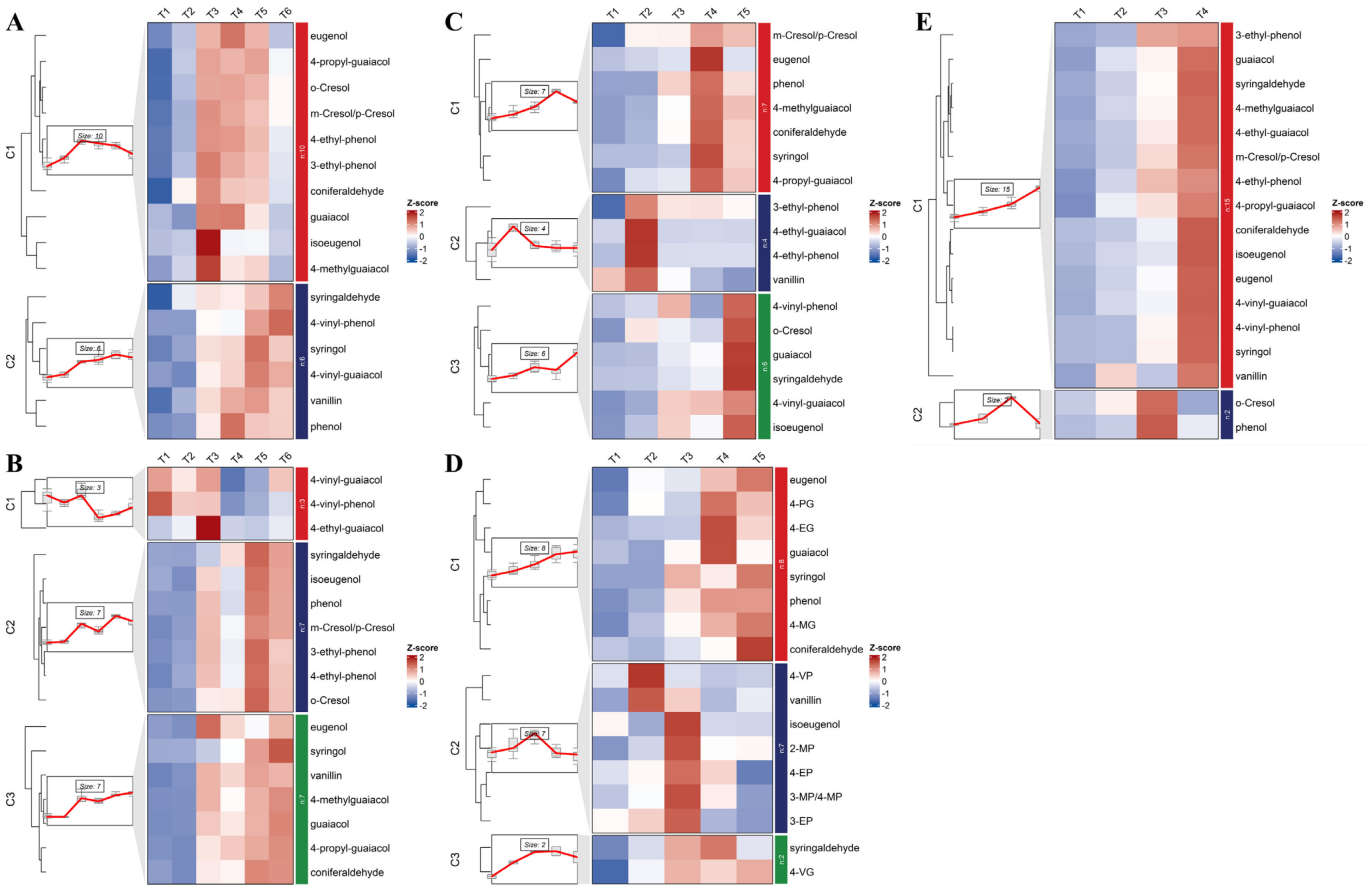
Time series analysis of total volatile phenols during the development of Cabernet Sauvignon from Ningxia **(A)**, Cabernet Sauvignon from Changli **(B)**, Shuanghong **(C)**, Tian grape **(D)**, and Yeniang No.2 **(E)**. (2-MP, *o*-cresol; 3-MP/4-MP, *m*/*p*-cresol; 4-EP, 4-ethyl-phenol; 3-EP, 3-ethyl-phenol; 4-MG, 4-methyl-guaiacol; 4-VP, 4-vinyl-phenol; 4-EG, 4-ethyl-guaiacol; 4-VG, 4-vinyl-guaiacol; 4-PG, 4-propyl-guaiacol).

Time-series analysis of free and bound volatile phenol concentrations during grape berry development was presented in **Figures 2** and **3**. In Cabernet Sauvignon, free volatile phenols consistently showed an initial increase followed by a subsequent decline. The peak concentrations occurred at the T3 stage in the Ningxia region, while in the Changli region, peaks were predominantly observed at T5. In contrast to *V. vinifera*, East Asian species exhibited significantly distinct developmental patterns of these volatile phenols. Coniferaldehyde and 4-vinyl-guaiacol displayed a pattern of progressive accumulation throughout berry development. The accumulation patterns of bound volatile phenols (**Figure 3**) closely mirrored those of total volatile phenol concentrations (free + bound) (**Figure 4**). Notably, the total volatile phenol levels better reflect the potential contribution of grape-derived compounds to wine aroma (**Figure 4**). In contrast to the free volatile phenols, total volatile phenols exhibited two discrete patterns that were distinctly influenced by different regions. In Cabernet Sauvignon from the Ningxia region, the predominant pattern involved a rapid increase to a peak at T3, followed by a gradual decline. This cluster included cresols, ethyl-phenols, guaiacol, 4-methyl-guaiacol, 4-propylguaiacol, eugenol, isoeugenol, and coniferaldehyde. Cabernet Sauvignon from the Changli region showed gradual increases across development (clusters 2 and 3). East Asian species displayed more progressive accumulation of total volatile phenols throughout berry development, especially in Yeniang No.2 and Tian grapes. In Tian grapes, eugenol, syringol, phenol, coniferaldehyde, guaiacol, and guaiacol’s alkyl derivatives showed a gradual increase. In contrast, vanillin, isoeugenol, and the cresols and ethyl-phenols showed an initial increase followed by a subsequent decrease. In Yeniang No. 2, most volatile phenols showed a consistent increasing trend, except for *o*-cresol and phenol, which exhibited an early peak followed by decline. East Asian grape species also showed a gradual increasing trend, similar to that of Cabernet Sauvignon from the Changli region, and consistent with previous studies on cresol, eugenol, syringol, guaiacol, and 4-methylguaiacol (Fenoll et al., 2009; Szeto et al., 2020; Noestheden et al., 2018).

It is noteworthy that high levels of bound volatile phenols were observed in the berries of East Asian species. The abundance of these glycosidically bound compounds suggests higher activity of glycosyltransferases (GTs) in these populations. Previous studies have found that several glycosyltransferases, *UGT72B27*, *UGT92G6* and several members of the *UGT85A* subfamily, are active in the glucosylation of multiple volatile phenols in grapes exposed to smoke (Härtl et al., 2017; Cao et al., 2023). In addition, certain volatile phenols, such as methyl eugenol, have been reported to deter herbivory or insect pests (Tan and Nishida, 2012). In this study, most volatile phenols began to accumulate before véraison, indicating that glycosyltransferase activity and substrate synthesis capacity are already active prior to véraison.

In summary, during grape development, certain volatile phenols exhibited notably high concentrations in specific East Asian species grapes (**Supplementary Table 6**). In particular, guaiacol, vanillin, 4-vinyl-guaiacol, 4-vinyl-phenol, and eugenol showed high concentrations in the Tian grapes whereas their levels in *V. vinifera* remained relatively low. The concentrations of multiple total volatile phenols in East Asian species showed progressive accumulation throughout berry maturation. Previous studies have also corroborated the high content of volatile phenols in East Asian species grape (Yang, 2024).

### 3.2 Identification of volatile phenols precursors

#### 3.2.1 The establishment of a volatile phenol precursor prediction library

Bound volatile phenols predominantly exist as glycosidic precursors in berries. The biosynthesis of these precursors involves diverse glycosyltransferases, which exhibit a distinct complement in different grape cultivars (Hewitt et al., 2024; van der Hulst et al., 2025). This enzymatic specificity provides a plausible explanation for the observed diversity in bound volatile phenol concentrations across varieties. Therefore, investigating the developmental dynamics of these phenolic precursors is essential for understanding their metabolic regulation during berry development. Previous research has analyzed specific glycosidic phenol, methylphenol, syringol, guaiacol, and methylguaiacol (Hayasaka et al., 2010a; van der Hulst et al., 2019), which are known to originate largely from wildfire. Nevertheless, the volatile phenol precursors derived from endogenous berry metabolism were relatively limited in number. The bound form of *m*/*p*-cresol, guaiacol, 4-vinyl-phenol, 4-vinyl-guaiacol, syringol, eugenol, isoeugenol, and coniferaldehyde were detected at high concentration in grapes. As the key endogenous precursors of 4-ethyl-phenol, 4-ethyl-guaiacol, syringol and eugenol have already been identified (Yang, 2024), this study focused on the identification of potential precursors for *m*/*p*-cresol, guaiacol, 4-vinyl-phenol, 4-vinyl-guaiacol, isoeugenol, and coniferaldehyde. Precursor screening was conducted using a previously reported pseudo-targeted metabolomic approach (Yang et al., 2025). The precursor library included the common glycosidic forms found in grapes: one monosaccharide model (H), and three disaccharide models: hexose-pentose (HP), dihexose (HH), and hexose-deoxyhexose (HDH). Based on the potential structures of these volatile phenols, the theoretical masses of different glycosidic forms were calculated. Due to the presence of formic acid in the mobile phase, the target compounds preferentially formed [M + COOH]^-^ adduct ions (Wei et al., 2021). Accordingly, chromatographic peaks of the predicted precursor compounds were extracted from all samples based on their [M + COOH]^-^ ion form. Comprehensive details regarding the final constructed prediction library are provided in **Supplementary Table 7**. A total of 112 candidate compounds were included in the predicted library, encompassing precursors of *m*/*p*-cresol (24), 4-vinylphenol (24), guaiacol (21), 4-vinylguaiacol (15), coniferaldehyde (7), and isoeugenol (21).

#### 3.2.2 Qualitative analysis of volatile phenol precursors

Extraction of predicted precursor peak areas based on library-matching of accurate mass using MS1 data. Pearson correlation analysis was conducted to evaluate the relationship between the increase in volatile phenols and the decrease of peak area in their predicted glycosidic precursors following hydrolysis in grape berries. Given that volatile phenol concentrations and glycosidic forms vary significantly across different grape varieties (Yang et al., 2025), some variety-specific precursors may be masked in correlation analyses based on a multi-varietal dataset. To minimize this confounding effect, comparative datasets were constructed by pairing *V*. *vinifera* samples exhibiting low volatile phenol levels with individual datasets from the additional species *V*. *quinquangularis*, *V*. *amurensis*, and *V*. *davidii*, respectively. Pearson correlations (r≥0.5) between hydrolytic aglycone release and glycoside depletion were used to select potential precursors. A total of 38 potential glycosidic precursors of volatile phenols were screened (**Table 1**). Although correlation analysis has its limitations and may overlook some precursors, it largely addresses the challenge of annotating the numerous complex peaks in full-scan data. At the same time, peaks showing high correlation based on hydrolysis data are clearly more likely to release free volatile phenols during winemaking, making them more relevant to wine production than uncorrelated or weakly correlated peaks. However, selection based solely on correlation also carries the risk of false positives. For further qualitative validation, secondary ion mass spectra (MS/MS) of the identified compounds were acquired.

**Table 1:** The correlation analysis of volatile phenol precursors in grape berries.

Volatile phenols	Precursors	Precise mass (m/z)	RT (min)	Correlation coefficient^a^
cresol	MP-H^b^	270.1104	2.46	0.92
			3.17	0.88
			7.5	0.87
	MP-HH	432.1632	2.22	0.79
			13.99	0.64
	MP-HDH	416.1683	5.9	0.70
			10.2	0.76
			13.9	0.75
	MP-HP	402.1526	3.84	0.75
			8.18	0.81
			9.64	0.88
			14.6	0.66
4-vinyl-phenol	VP-H	282.1104	6.92	0.54
			10.38	0.74
			11.25	0.84
	VP-HH	444.1632	15.04	0.68
guaiacol	GU-HDH	432.1632	14.98	0.83
			2.25	0.96
			13.99	0.87
	GU-HP	418.1475	13.5	0.52
			14.04	0.53
4-vinyl-guaiacol	VG-H	312.1210	14.63	0.93
	VG-HH	474.1738	3.22	0.70
			15.09	0.57
	VG-HDH	458.1789	14.65	0.79
	VG-HP	444.1632	1.74	0.57
			15.03	0.72
isoeugenol	IEU-H	326.1366	10.85	0.81
			11.47	0.60
			11.82	0.67
	IEU-HH	488.1894	2.9	0.80
			6.5	0.54
			14.74	0.86
	IEU-HDH	472.1945	3.13	0.94
			3.68	0.79
	IEU-HP	458.1788	14.65	0.71
coniferaldehyde	COA-HH	502.1687	14.73	0.71
	COA-HDH	486.1738	2.07	0.56

a Correlation analysis of bound volatile phenols and the reduction of potential precursors were performed using Pearson’s method.

b MP, cresol; VP, 4-vinyl-phenol; GU, guaiacol; VG, 4-vinyl-guaiacol; IEU, isoeugenol; COA, coniferaldehyde; H, hexose; HH, dihexose; HP, hexose-pentose; HDH, hexose-deoxyhexose.

Secondary ion mass spectral (MS/MS) fragments of precursors exhibiting strong correlations were collected for qualitative analysis. Identification of precursors was achieved by comparing the MS/MS spectra with the theoretical fragment masses calculated from the structures of precursors. In addition, CFM-ID was used to predict some fragment ions and provided proposed fragmentation pathways (Wang et al., 2021). Based on MS/MS analysis, a total of 20 potential glycosylated volatile phenol precursors were identified (**Supplementary Figures S1**-**S6**), with their retention times and characteristic fragments summarized in **Table 2**. In this study, characteristic fragment ions at m/z 101.02, 113.02, 119.03, 125.02, and 143.03 were frequently observed. These fragments were recognized as sugar moieties in glycosides reported by a previous study (Caffrey et al., 2019). The precursor ions identified in this study, including m/z 191.05, 269.11, and 415.16 for *m*/*p*-cresol precursors, as well as m/z 149.10 (corresponding to 4-vinyl-guaiacol-hexoside) and 177.04 (corresponding to coniferaldehyde-dihexoside), were consistent with the fragment ions previously reported in the literature (Barnaba et al., 2018; Caffrey et al., 2019).

**Table 2:** Qualitative results of key volatile phenol precursors by target MS/MS.

Forms	Theoretical mass (m/z)	Compounds	RT (min)	MS/MS fragments (m/z)
VP-H^a^	282.1104	VP-H 6.92	6.92	112.98, 119.05, 135.04, 149.01, 178.02, 193.05, 195.05, 251.54
		VP-H 10.38	10.38	121.07, 125.19, 165.06, 177.10, 191.03
		VP-H 11.25	11.25	103.00, 135.04, 165.02, 180.03, 193.05
VP-HH	444.1632	VP-HH 15.04	15.04	101.02, 125.02, 179.11, 161.04, 191.11, 327.22, 371.74, 443.19
MP-H	270.1104	MP-H 2.46	2.46	107.01, 119.03, 135.04, 149.06
		MP-H 7.50	7.50	123.04, 153.02, 269.11
MP-HDH	416.1683	MP-HDH 13.94	13.94	101.02, 113.02, 119.03, 125.02, 143.03, 149.04, 161.04, 191.05, 415.16
MP-HP	402.1526	MP-HP 9.64	9.64	113.02, 119.03, 125.02, 165.05, 239.09, 285.04, 339.05, 387.09
		MP-HP 14.60	14.60	113.04, 133.01, 161.02, 285.04, 298.98, 342.18, 359.20
GU-HDH	432.1632	GU-HDH 13.99	13.99	119.03, 149.01, 165.01, 183.06, 255.11, 279.06, 284.03, 327.07 357.13
		GU-HDH 14.98	14.98	101.02, 120.99, 207.17, 269.18, 345.10, 353.08, 359.24, 415.27, 447.26
GU-HP	418.1475	GU-HP 14.40	14.40	101.02, 133.03, 177.02, 255.03, 280.75, 300.03
VG-H	312.1210	VG-H 14.63	14.63	121.25, 125.02, 149.10, 191.08, 195.06
VG-HH	474.1738	VG-HH 15.10	15.10	101.03, 119.05, 145.03, 163.04, 179.05, 358.12, 459.24, 473.28, 489.27
COA-HH	502.1687	COA-HH 14.73	14.73	101.02, 113.02, 119.03, 125.02, 161.05, 177.04, 251.13, 269.14, 385.23, 431.19, 501.23
COA-HDH	486.1738	COA-HDH 2.07	2.07	101.02, 113.02, 119.03, 147.06, 219.03, 353.14, 469.15, 48518
IEU-H	326.1366	IEU-H 10.85	10.85	101.02, 119.04, 147.08, 163.08, 191.07, 209.08, 265.25
IEU-HH	488.1894	IEU-HH 2.90	2.90	165.46, 191.02, 204.27, 255.08
IEU-HDH	472.1945	IEU-HDH 3.13	3.13	149.01, 193.01, 355.07, 411.08, 473.08
		IEU-HDH 3.68	3.68	137.02, 165.02, 193.01, 355.06, 397.07

a MP, cresol; VP, 4-vinyl-phenol; GU, guaiacol; VG, 4-vinyl-guaiacol; IEU, isoeugenol; COA, coniferaldehyde; H, hexose; HH, dihexose; HP, hexose-pentose; HDH, hexose-deoxyhexose.

### 3.3 Concentration and developmental dynamics of volatile phenol precursors in grapes

#### 3.3.1 The volatile phenol precursors in ripen grapes

The analysis of key volatile phenol precursors in grape berries (**Supplementary Table 8**) included 20 newly identified precursors from this work (**Table 2**), in addition to the previously characterized precursors of 4-ethyl-phenol, 4-ethyl-guaiacol, eugenol, and syringol (**Supplementary Table 4**). The sample data were compared based on peak areas (Ghaste et al., 2015). Among these, the three precursors of 4-vinyl-phenol (VP-H 6.92, VP-H 11.25, and VP-HH 15.04) exhibited the highest concentrations in Tian grapes. Correspondingly, the total concentration of 4-vinyl-phenol was also markedly higher in Tian grapes compared with the other four varieties. The precursor VG-H 14.63 of 4-vinyl-guaiacol reached its highest concentration in Yeniang No.2, while VG-HH 15.10 showed maximal concentrations in both Yeniang No.2 and Tian grapes consistent with the significantly elevated concentrations of 4-vinylguaiacol observed in these two cultivars. Similar accumulation patterns were observed for guaiacol, isoeugenol, and coniferaldehyde. The highest concentrations of EU-H 14.2 and EU-HP 14.6 were observed in the Yeniang No.2, which agrees with the findings recently reported by Yang et al. (2025).

The glycosidic forms of *p*-coumaric acid and ferulic acid, known precursors of 4-ethyl-phenol and 4-ethyl-guaiacol (Yang, 2024), require microbial metabolic conversion during wine fermentation (Milheiro et al., 2019). As a result, these glycosidic precursors do not directly correlate with their corresponding volatile phenols in grape berries. Marked varietal differences were observed in the accumulation of glycosidic *p*-coumaric acid and ferulic acid. Yeniang No.2 contained the highest concentrations of Cou-H 11.6, Fer-H 8.7, and Fer-H 9.1, whereas Shuanghong showed predominant accumulation of Cou-HH 2.8. The Tian grapes showed notable enrichment in several precursors including Cou-H 1.6, Cou-H 6.7, Cou-H 9.1, Cou-HP 5.1, Fer-H 1.8, Fer-H 9.1, Fer-HH 3.2, and Fer-HP 5.6. Cabernet Sauvignon from two regions primarily accumulated the highest levels of Cou-H 4.4 and Fer-HH 3.8.

Mature berries exhibited pronounced interspecific diversity in volatile phenol precursor profiles. Tian grapes contained the highest levels of precursors for most *m*/*p*-cresol, 4-vinyl-phenols, guaiacols, and ferulic acid, which aligns directly with their elevaated volatile phenol levels (Section 3.1.1). Moreover, Shuanghong and Yeniang No.2 showed no significant differences in free guaiacol concentrations, yet their precursor profiles diveraged markedly: Yeniang No.2 accumulated higher level of GU-HP 14.40, whereas Shuanghong was predominated by GU-HDH 13.99. This demonstrates that precursor profiling can reveal varietal differences that are masked when examining only aglycones (Ghaste et al., 2015).

The high concentration of glycosidically bound volatile phenols in East Asian species likely reflects activation of a detoxification and defense mechanism. In response to smoke exposure, grapes glycosylate exogenous volatile phenols to mitigate toxicity (Hewitt et al., 2024). We propose that East Asian species activate a similar stress-response pathway in response to their high endogenous accumulation of volatile phenols, resulting in elevated precursor levels.

#### 3.3.2 Evolution of key volatile phenol precursors in grape development

**Figure 5:**
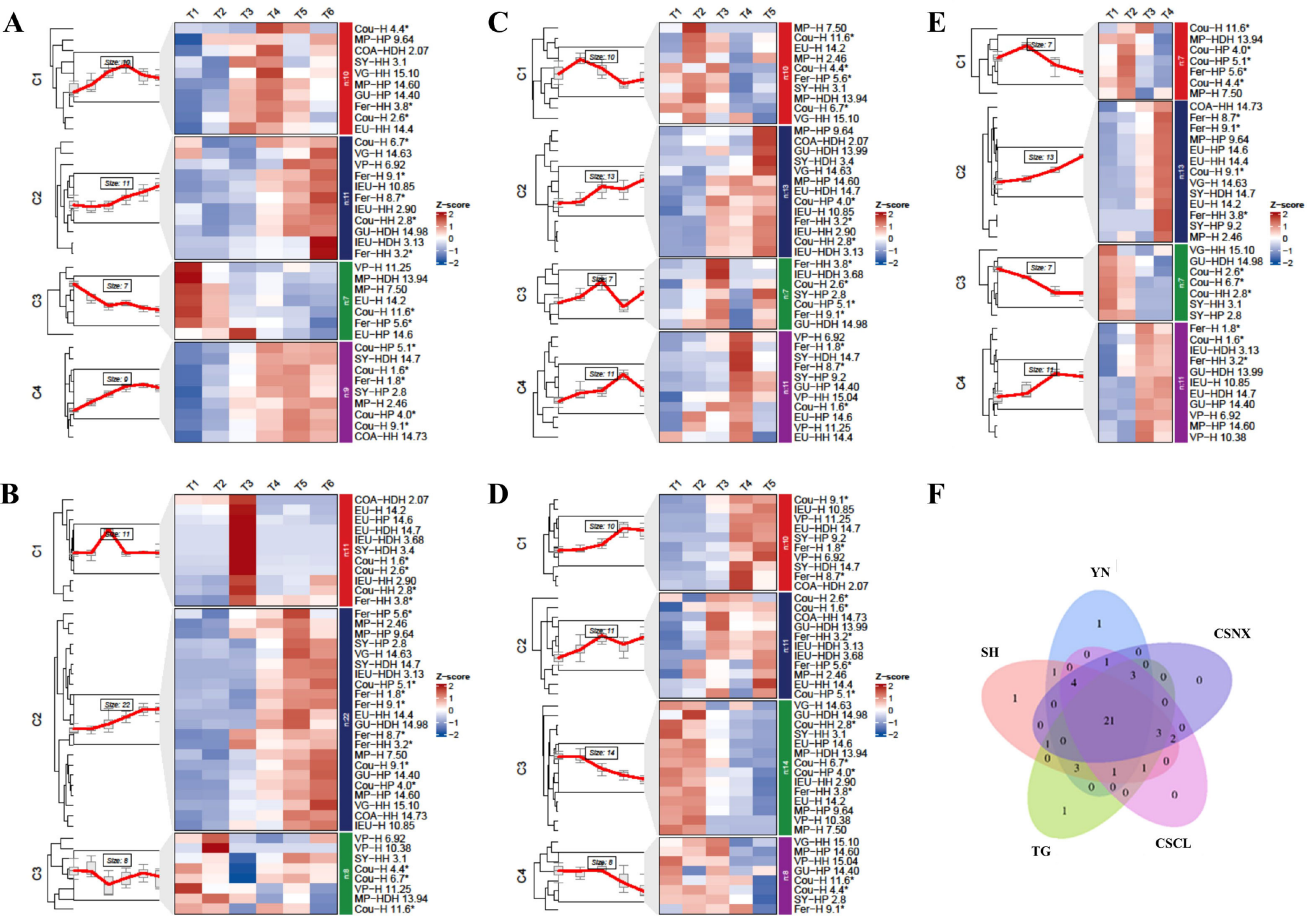
Time series analysis of key volatile phenol precursors during the development of Cabernet Sauvignon from Ningxia **(A)**, Cabernet Sauvignon from Changli **(B)**, Shuanghong **(C)**, Tian grape **(D)**, Yeniang No.2 **(E)**, and distribution of key precursors in different varieties of ripe grapes **(F)**; The precursors marked with an asterisk (*) are those that require microbial metabolism to form volatile phenols. (Cou, p-Coumaric acid; Fer, ferulic acid; EU, eugenol; SY, syringol; MP, cresol; VP, 4-vinyl-phenol; GU, guaiacol; VG, 4-vinyl-guaiacol; IEU, isoeugenol; COA, coniferaldehyde.

To further elucidate how the volatile phenol precursors accumulate during berry development, a time-series analysis was performed to investigate their developmental dynamics across different grape varieties (**Figure 5**), focusing on identified glycosidic precursors. Tian grapes and Yeniang No.2 exhibited two main diversity patterns: one involving progressive accumulation and the other a gradual decrease. Notably, different precursors of the same volatile phenol displayed opposing developmental trends, possibly due to interconversion during grape development. Similar observations were reported by Pardo-García et al. (2017) in their study on glycosylated guaiacol derivatives. Some of these precursors show changes that are correlated with those of volatile phenols. Those precursors can serve as reliable indicators for reflecting the changes in these compounds within the berry and predicting the volatile phenol levels in the resulting wine (Kennison et al., 2008). Consistent with previous findings, this study observed similar trends. Despite divergent accumulation patterns of volatile phenols across the grape varieties analyzed, some of the precursors identified herein exhibited change trends congruent with their respective aglycones. Specifically, the *m*/*p*-cresol precursors MP-HP 9.64 and MP-HP 14.60 mirrored the overall concentration dynamics of *m*/*p*-cresol in the berries. Likewise, the isoeugenol precursors IEU-H 10.85 and IEU-HDH 3.13, as well as the coniferaldehyde precursors COA-HDH 2.07 and COA-HH 14.73, displayed temporal patterns analogous to those of isoeugenol and coniferaldehyde, respectively (**Supplementary Figure 7**). These precursors were widely detected across grape cultivars, suggesting their ubiquity in grape metabolism. Consequently, they held broader application potential for predicting the dynamics of *m*/*p*-cresol, isoeugenol, and coniferaldehyde during fruit development.

A substantial proportion of volatile phenol precursors exhibited a pattern of gradual accumulation during berry development across all investigated cultivars, aligning with the earlier findings of Szeto et al. (2024). This pattern supports the hypothesis that glycosylation serves as a detoxification mechanism, stabilizing volatile phenols by sequestering them in vacuoles to prevent interference with cellular physiology (Hewitt et al., 2024; Song et al., 2018).

Regional influence on the evolution of these precursors were evident: In the Ningxia region, glycosidic precursors of *p*-coumaric acid and isoeugenol exhibited a gradual increase during the early developmental stages. In contrast, in the Changli region, only Cou-HP 4.0, Cou-H 9.1, and IEU-H 10.85 demonstrated a similar accumulation trend.

Varietal differences emerged through both shared and unique precursor profiles. As illustrated in **Figure 5F**, 21 volatile phenol precursors were detected in all five mature grape cultivars. A part of them such as MP-H 2.46, MP-HP 9.64, VP-H 6.92, GU-HP 14.40, IEU-HDH 3.13, EU-HH 14.4, EU-HP 14.6, and Cou-H 6.7 exhibited particularly high responses in the samples (**Supplementary Table 8**). These findings confirm that even uncontaminated *Vitis vinifera* wines contain volatile phenols derived from endogenous precursors (Coulter et al., 2022).

Species-specific signatures were more distinctive. The precursors GU-HDH 13.99, EU-HDH 14.7 and SY-HP 9.2 were consistently detected in all three East Asian species but absent in *V. vinifera*, likely contributing to the elevated guaiacol, eugenol, and syringol levels in these East Asian cultivars. In Shuanghong, a specific precursor (SY-HDH 3.4) showed gradual increase over development. Unique detection of VP-H 10.38 and IEU-HDH 3.68 was noted in mature Yeniang No.2 and Tian grapes, respectively.

Cultivar-specific developmental trajectories further distinguished the varieties. In the Tian grapes, the majority of *m*/*p*-cresol precursors, with the notable exception of MP-H 2.46, declined progressively during berry development. Concurrently, the Yeniang No.2 exhibited a gradual accumulation of eugenol precursors. In contrast, these specific precursors demonstrated opposing or different trends in the other varieties. These divergent trends underscore how distinct glycosyltransferase activities and substrate availabilities shape the unique metabolic phenotypes of each cultivar.

Interspecific diversity in volatile phenol precursor profiles reflects the interplay of three key factors. First, grape species differ inherently in their capacity for volatile phenol biosynthesis, which directly influence substrate availability and the intensity of the glycosylation-meactivated detoxification response. Second, cultivar-specific expression patterns of glycosyltransferases (Härtl et al., 2017; van der Hulst et al., 2025) shape distinct precursor spectra, as these enzymes exhibit unique substrate preferences and catalytic efficiencies. Third, tissue-specific compartmentalization and potential translocation from vegetative organs (Hayasaka et al., 2010b; Sousa et al., 2020) further modulate precursor pools within the berry.

Although the complete metabolic pathways of glycosidically-bound volatile phenols remain to be fully elucidated, our developmental time-series data nonetheless provide fundamental insights into the evolutionary mechanisms driving interspecific metabolic diversification in grapes (Yang et al., 2025).

## 4 Conclusion

This study establishes that East Asian grape species (*V. davidii*, *V. quinquangularis*, *V. amurensis*) operate as constitutive high-volatile phenol systems, progressively accumulating these compounds from pre-véraison through maturity—unlike *V. vinifera*, which shows lower and more variable accumulation. Using a pseudo-targeted metabolomic approach, we identified 20 glycosidic precursors, including six species-specific conjugates (GU-HDH 13.99, EU-HDH 14.7, SY-HP 9.2, SY-HDH 3.4, VP-H 10.38, IEU-HDH 3.68) detected exclusively in East Asian species, providing clear evidence of lineage-specific glycosyltransferase specialization. Furthermore, broadly distributed precursors such as MP-HP 9.64 (*m/p*-cresol), IEU-HDH 3.13 (isoeugenol), and COA-HH 14.73 (coniferaldehyde) co-accumulated with their corresponding volatile phenols, demonstrating predictive potential for estimating aroma potential across cultivars.

These findings have practical implications: (1) grape breeding can target glycosyltransferase alleles to modulate aroma profiles; (2) viticulture must consider that early-season conditions influence pre-véraison volatile phenol metabolism; and (3) winemaking strategies can be tailored to species-specific precursor signatures, such as optimizing enzymatic release for East Asian wines versus minimizing extraction for smoke-sensitive *V. vinifera*.

## Data Availability

The raw data supporting the conclusions of this article will be made available by the authors, without undue reservation.
