# Supplementary material for: Diversity of volatile phenols and their newly identified precursors during different grape species development
Source: Front Plant Sci. 2026 Mar 11;17:1746115. doi: 10.3389/fpls.2026.1746115 (PMC13013403; doi:10.3389/fpls.2026.1746115)
Supplement: Supplementary file 1 [file DataSheet1.docx]

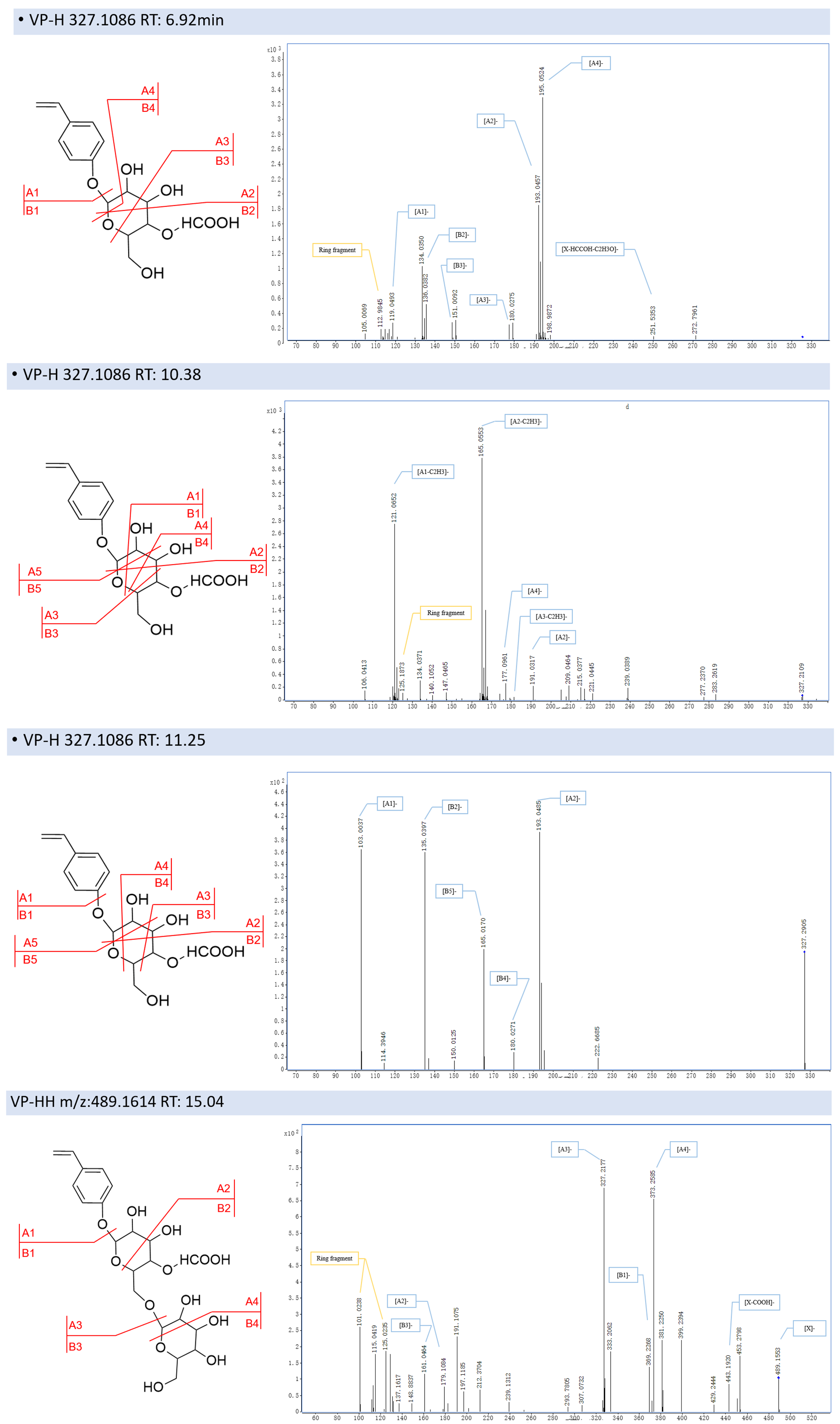


# Figure S1 Analysis of MS2 spectra of 4-vinyl-phenol precursors.

**
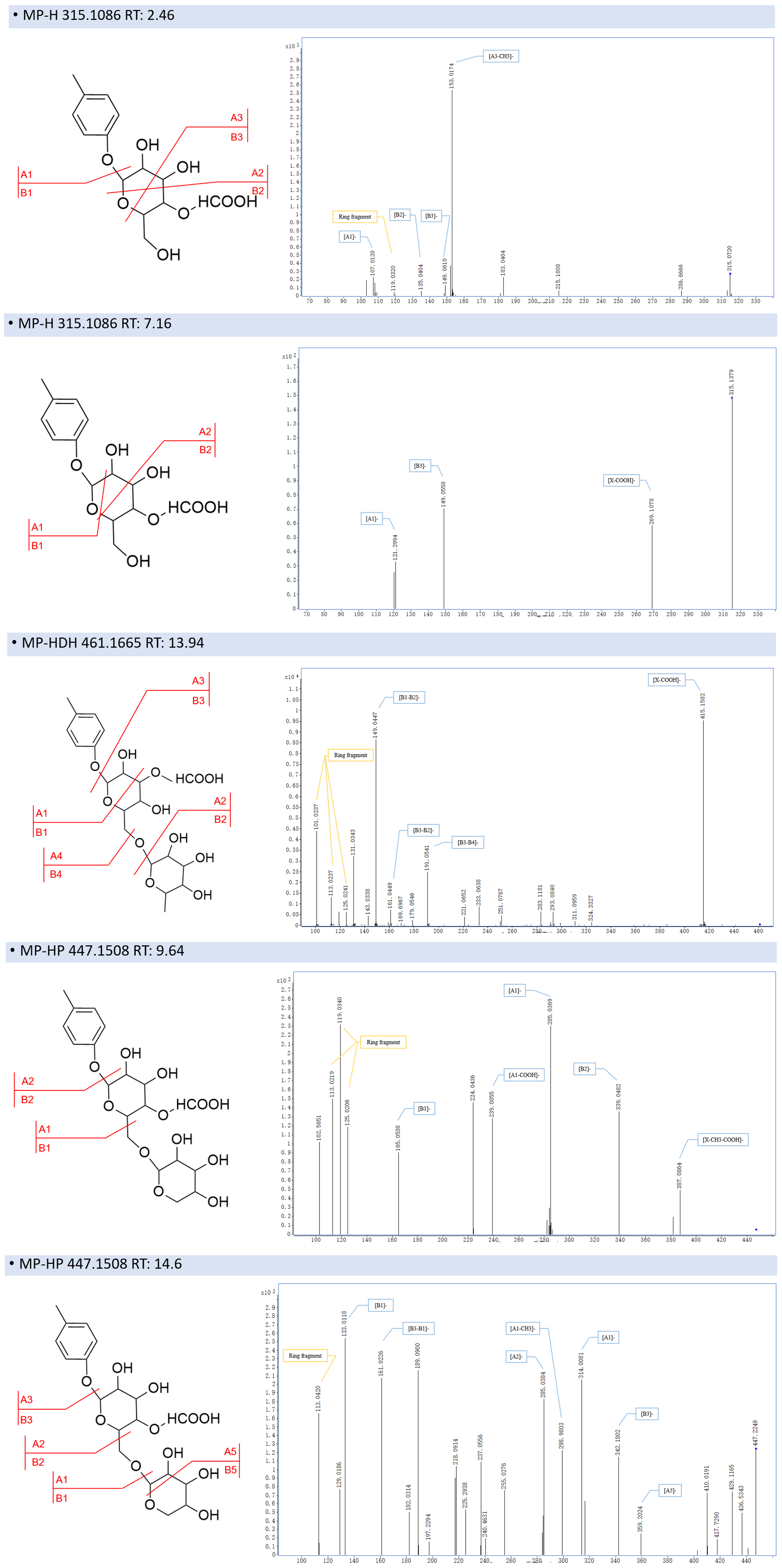
**

# Figure S2 Analysis of MS^2^ spectra of cresol precursors.


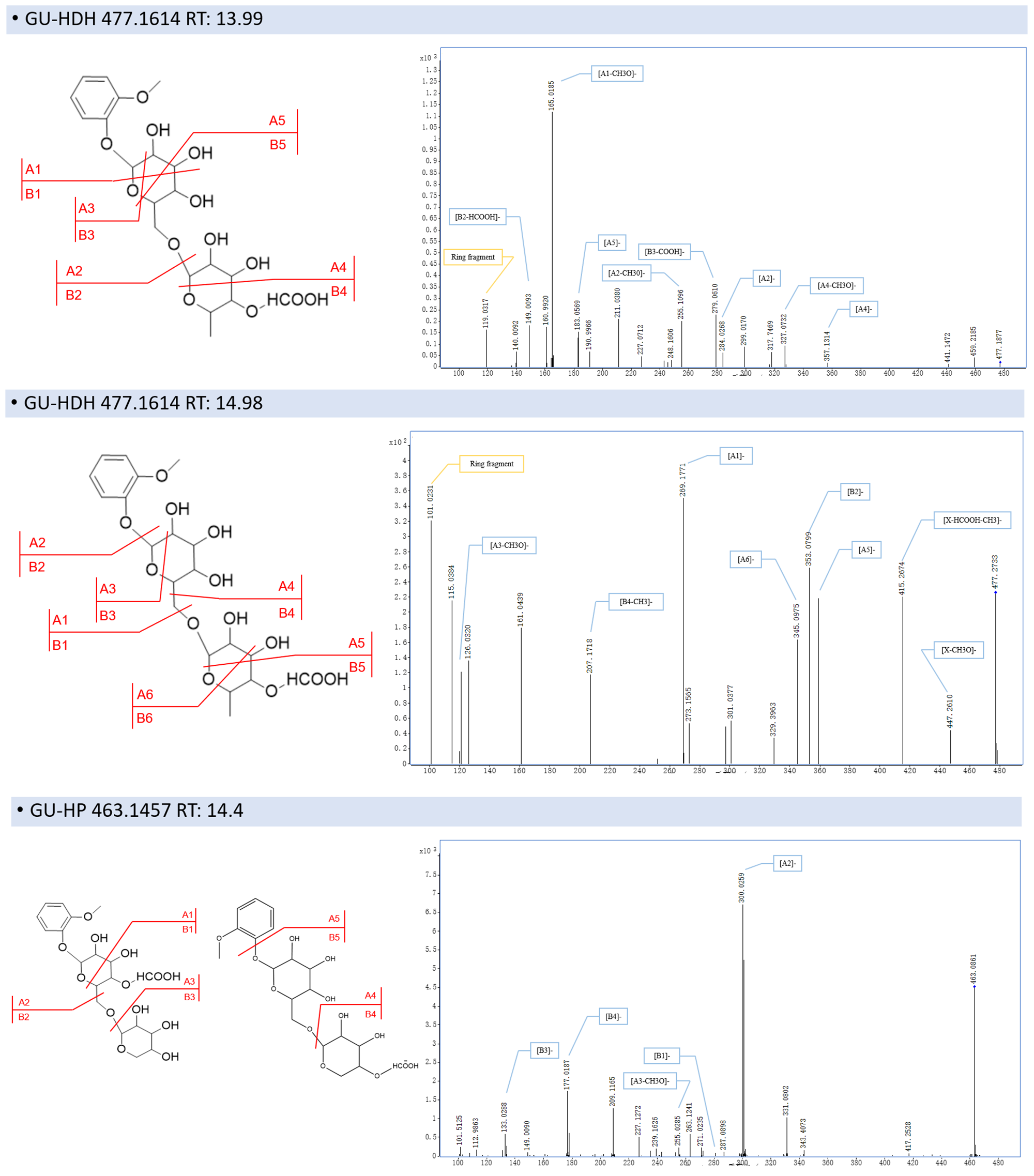


# Figure S3 Analysis of MS^2^ spectra of guaiacol precursors.


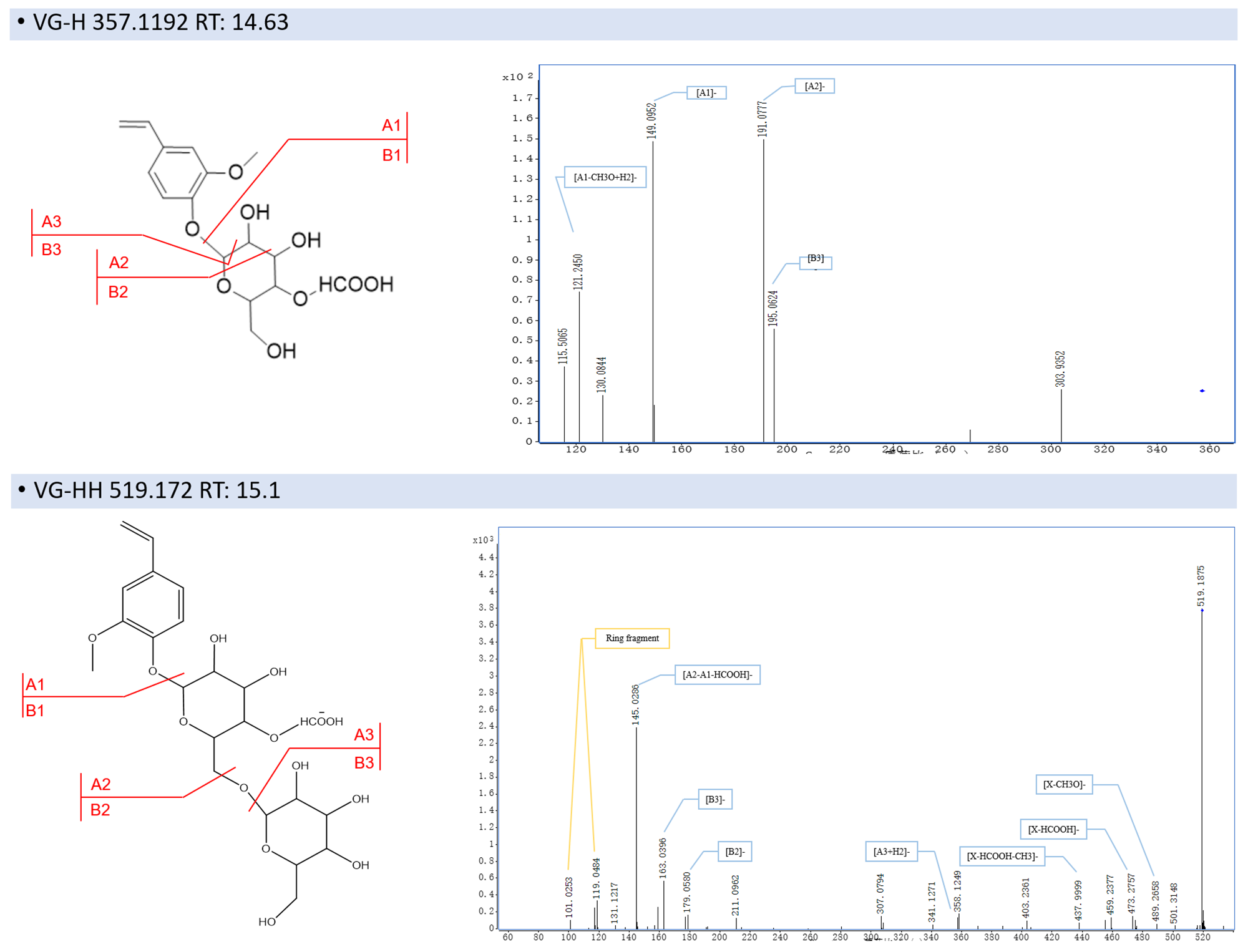


# Figure S4 Analysis of MS^2^ spectra of 4-vinyl-guaiacol precursors.


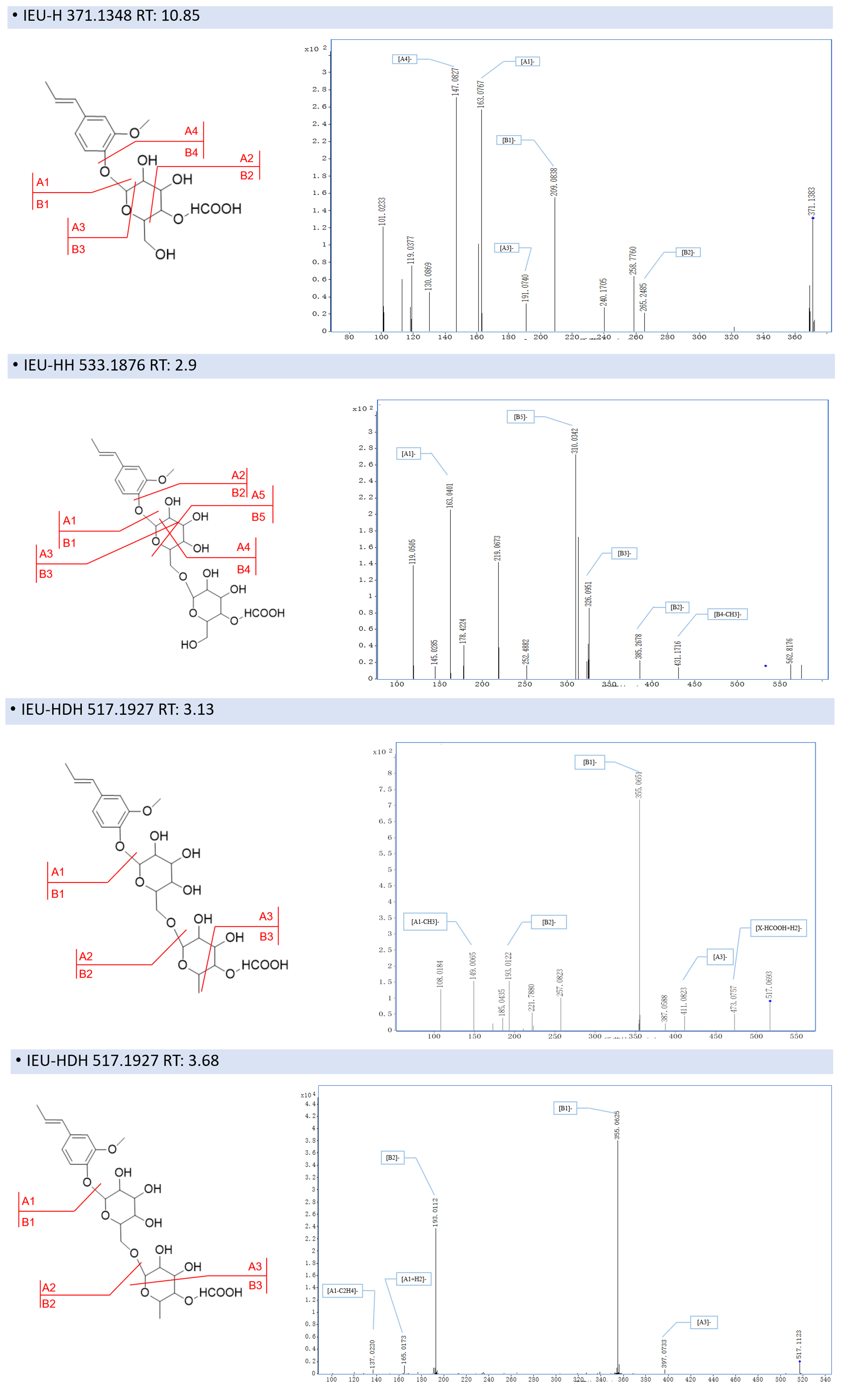


# Figure S5 Analysis of MS^2^ spectra of isoeugenol precursors.


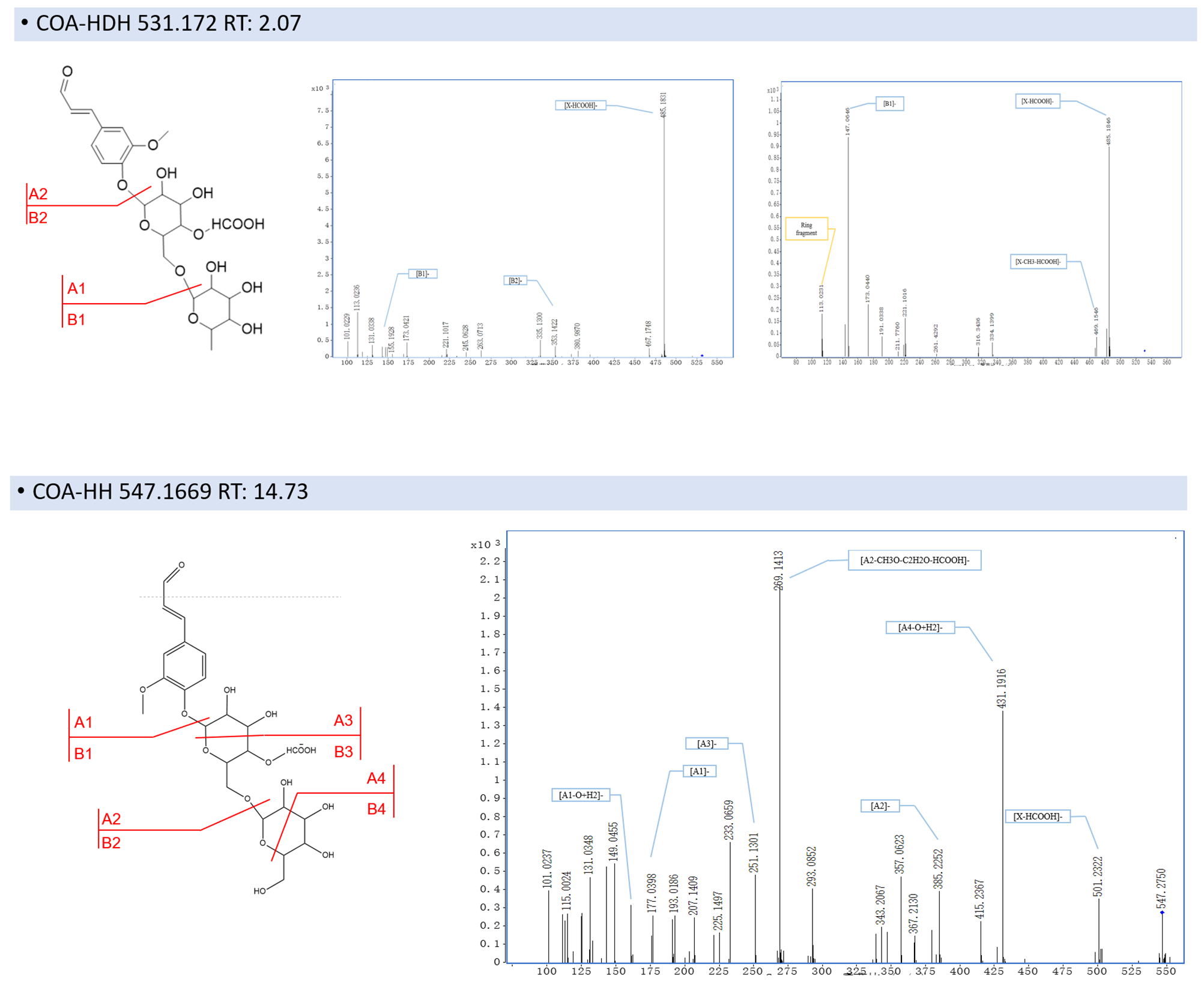


# Figure S6 Analysis of MS2 spectra of coniferaldehyde precursors.


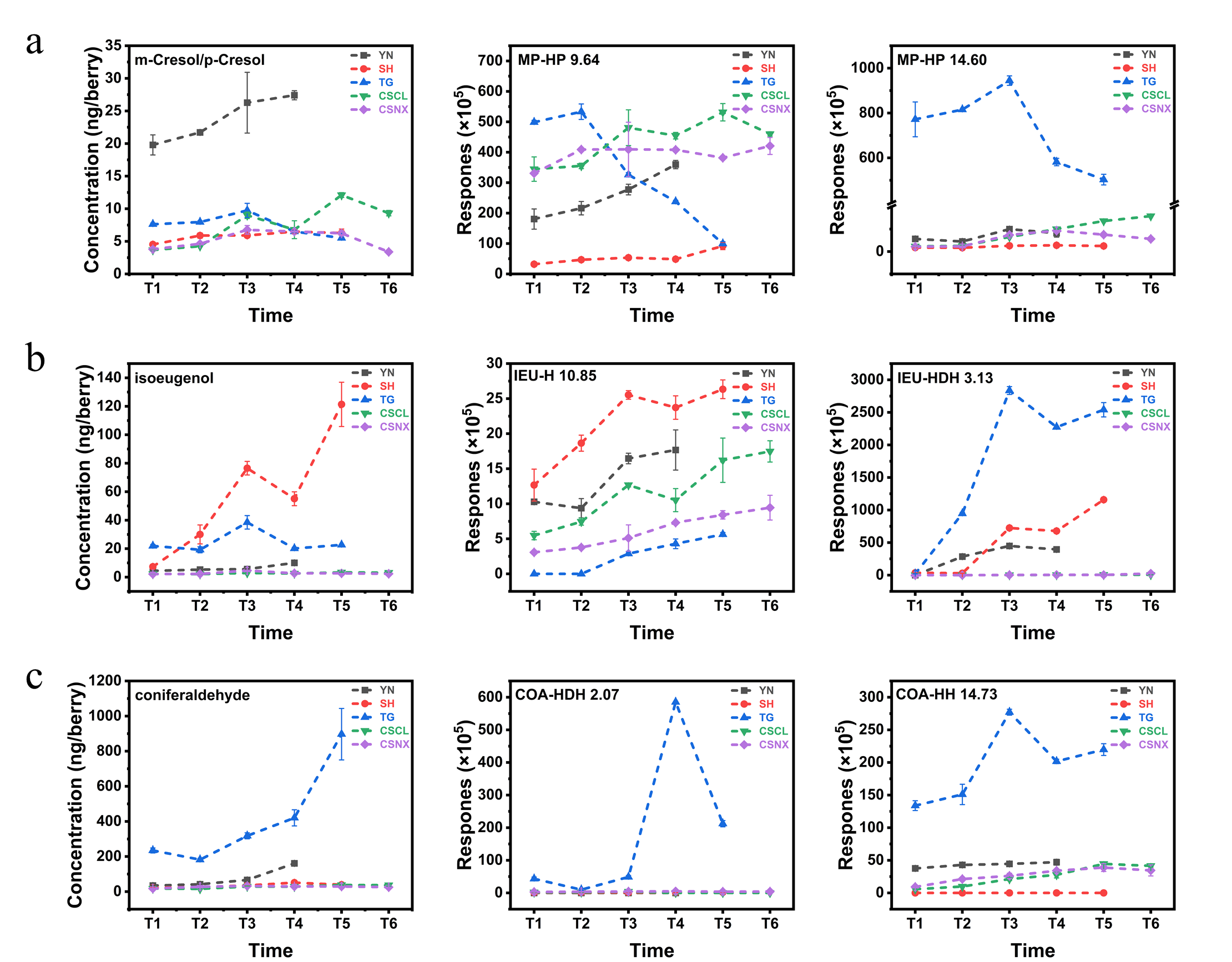


# Figure S7 Changes in the total volatile phenols and their precursors; a, *m*/*p*-cresol; b, isoeugenol; c, coniferaldehyde; CSNX, Cabernet Sauvignon from Ningxia, CSCL, Cabernet Sauvignon from Changli, SH, Shuanghong, TG, Tian grape, YN, Yeniang No.
